# USEFUL FIELD OF VIEW AND DRIVING SIMULATION ACROSS TWO AGE GROUPS

**DOI:** 10.1093/geroni/igz038.3511

**Published:** 2019-11-08

**Authors:** Rebecca A Dunterman, Robert C Intrieri, Marisa Guernsey

**Affiliations:** 1 Auburn University, Auburn, Alabama, United States; 2 Western Illinois University, Macomb, Illinois, United States

## Abstract

The Insurance Information Institute (2017) reports that drivers aged 65 and older have the second highest rate of fatal car crashes. Research with the useful field of view (UFOV) assessment has predicted crashes in older drivers (Ball, 2006). “UFOV is defined as the area from which an individual can extract information quickly without head or eye movement” (Posit Science, 2019). Research demonstrates that older drivers are limited by poorer vision, divided attention and the inability to ignore distractions, and slower reaction time to critical stimuli (Owsley et al. 1998). As a result UFOV is an effective variable in assessing driver safety. We hypothesized that older compared to younger drivers would be less likely to inhibit attention to task irrelevant visual stimuli while engaged in a simulated driving task. Participants were community dwelling older adults and students recruited from a research pool and through word of mouth. Participants completed a series of demographic and health questions, Snellen visual acuity test a series of cognitive measures (e. g., Trails 1 and 2, digit symbol, digit span) and the UFOV assessment. Participants completed a driving simulation task while information on driving performance: number of collisions, speed limit deviations, turn signal use, time spent tailgating another vehicle, and braking reaction times. ANOVA demonstrated that as hypothesized, younger participants had significantly lower UFOV risk scores (p = .000). Older adults’ (M = 2.15, SD = .945) and younger adults’ (M = 1, SD = 0).

